# Combining Network Pharmacology and Experimental Validation to Study the Action and Mechanism of Water extract of Asparagus Against Colorectal Cancer

**DOI:** 10.3389/fphar.2022.862966

**Published:** 2022-06-14

**Authors:** Huiling Liang, Yanju Li, Feiqing Wang, Jianing Zhao, Xu Yang, Dan Wu, Chike Zhang, Yanqing Liu, Jie Huang, Min Su, Zhixu He, Yang Liu, Jishi Wang, Dongxin Tang

**Affiliations:** ^1^ Department of Scientific Research, The First Affiliated Hospital of Guizhou University of Traditional Chinese Medicine, Guiyang, China; ^2^ Department of Hematology, Affiliated Hospital of Guizhou Medical University, Guiyang, China; ^3^ Academy of Medical Engineering and Translational Medicine, Medical College of Tianjin University, Tianjin, China; ^4^ National and Guizhou Joint Engineering Laboratory for Cell Engineering and Biomedicine Technique, Guizhou Province Key Laboratory of Regenerative Medicine, Key Laboratory of Adult Stem Cell Translational Research, Chinese Academy of Medical Sciences, Guizhou Medical University, Guiyang, China

**Keywords:** colorectal cancer, Chinese medicine, network pharmacology, asparagus, PI3K/AKT/mTOR signaling pathway

## Abstract

*Asparagus* (ASP) is a well-known traditional Chinese medicine with nourishing, moistening, fire-clearing, cough-suppressing, and intestinal effects. In addition, it exerts anti-inflammatory, antioxidant, anti-aging, immunity-enhancing, and anti-tumor pharmacological effect. The anti-tumor effect of ASP has been studied in hepatocellular carcinoma. However, its action and pharmacological mechanism in colorectal cancer (CRC) are unclear. The present study aimed to identify the potential targets of ASP for CRC treatment using network pharmacology and explore its possible therapeutic mechanisms using *in vitro* and *in vivo* experiments. The active compounds and potential targets of ASP were obtained from the TCMSP database, followed by CRC-related target genes identification using GeneCards and OMIM databases, which were matched with the potential targets of ASP. Based on the matching results, potential targets and signaling pathways were identified by protein-protein interaction (PPI), gene ontology (GO) functions, and Kyoto Encyclopedia of Genes and Genomes (KEGG) pathway enrichment analyses. Finally, *in vitro* and *in vivo* experiments were performed to further validate the anti-cancer effects of ASP on CRC. Network pharmacology analysis identified nine active components from ASP from the database based on oral bioavailability and drug similarity index, and 157 potential targets related to ASP were predicted. The PPI network identified tumor protein 53 (TP53), Fos proto-oncogene, AP-1 transcription factor subunit (FOS), and AKT serine/threonine kinase 1 (AKT1) as key targets. GO analysis showed that ASP might act through response to wounding, membrane raft, and transcription factor binding. KEGG enrichment analysis revealed that ASP may affect CRC through the phosphatidylinositol-4,5-bisphosphate 3-kinase PI3K/AKT/mechanistic target of rapamycin kinase (mTOR) signaling pathway. *In vitro*, ASP inhibited cell proliferation, migration, and invasion of HCT116 and LOVO cells, and caused G0/G1 phase arrest and apoptosis in CRC cells. *In vivo*, ASP significantly inhibited the growth of CRC transplanted tumors in nude mice. Furthermore, pathway analysis confirmed that ASP could exert its therapeutic effects on CRC by regulating cell proliferation and survival through the PI3K/AKT/mTOR signaling pathway. This study is the first to report the potential role of ASP in the treatment of colorectal cancer.

## 1 Introduction

Colorectal cancer (CRC) is a highly prevalent malignancy of the digestive system and is the second leading cause of cancer-related deaths worldwide, with a higher incidence in men than in women, and has the third highest rate of malignant lethality (Zhu et al., 2017). Approximately 20%−25% of patients with CRC already have metastases at the time of initial diagnosis because of the insidious onset of colorectal cancer and the lack of effective clinical screening biomarkers (Xu et al., 2020). One study predicted that by 2030, there will be more than 2.2 million new cases and 1.1 million cancer deaths worldwide. The burden will increase by 60%, with low- and middle-income countries facing greater challenges (Arnold et al., 2017). In recent years, good progress has been made in surgical techniques and systemic drug therapy; however, side effects, such as nausea, vomiting, pain, and leukopenia, can be particularly distressing for patients with CRC (Hattori et al., 2019; Vodenkova et al., 2020). Encouragingly, traditional Chinese medicine (TCM) has played an increasingly important role in the treatment of CRC as a major source of natural medicines and herbal products, with advantages of reliable efficacy and low rates of adverse effects.

In recent years, several studies have described the extensive use of TCM in cancer treatment, which has received increasing international attention (Chen et al., 2018; Wang et al., 2018). In a cohort study including 1,988 patients with lung adenocarcinoma treated with tyrosine kinase inhibitors (TKI), TCM treatment was associated with better progression-free survival and overall survival compared with those of the untreated group (Li C. L. et al., 2019). Similarly, in CRC, a multicenter prospective cohort study including 312 patients with stage II and III CRC reached similar conclusions (Xu et al., 2017). In a retrospective cohort study including 817 patients with CRC, TCM improved disease-free survival in patients with stage II and III disease (Shi et al., 2017). With the development of herbal injections, the intravenous delivery of antitumor herbs has been used widely in clinical practice. A study containing 366 randomized controlled trials and 48 systematic evaluations and meta-analyses reported more consistent benefits of herbal injections in terms of tumor response, quality of life, bone marrow suppression, and improvement in immune function (Yang et al., 2021). It was found that the active ingredients of herbal medicines usually exert their anti-cancer effects by inhibiting cell proliferation, inducing apoptosis, suppressing metastasis, inhibiting angiogenesis, reversing multidrug resistance, and modulating immune function (Yan et al., 2017; Li X. et al., 2019; Liu et al., 2020).

Chinese asparagus (*Asparagus cochinchinensis* (Lour) Merr., hereafter referred to as ASP) is a tuberous root vegetable in the lily family, which is widely distributed in Asia and is one of the common Chinese herbs with a long history in China (Lee et al., 2017). ASP has nourishing, moistening, fire-clearing, cough-suppressing, and intestinal effects and exerts anti-inflammatory, antioxidant, anti-aging, immunity-enhancing, and anti-tumor pharmacological effect (Samad et al., 2014; Lei et al., 2017; Cheng et al., 2019; Sun et al., 2020). Recent studies have reported that ASP polysaccharides can inhibit hypoxia-induced hepatocellular carcinoma cell migration, invasion, and angiogenesis by regulating hypoxia inducible factor alpha (HIF-1α) and vascular endothelial growth factor (VEGF) expression through mitogen activated protein kinase (MAPK) and phosphatidylinositol-4,5-bisphosphate 3-kinase (PI3K) signaling pathways (Cheng et al., 2021). In addition, ASP-derived exosome-like nanovesicles could inhibit hepatocellular carcinoma cell proliferation, induce apoptosis, and inhibit tumor growth without significant side effects in mice (Zhang et al., 2021).

**FIGURE 1:**
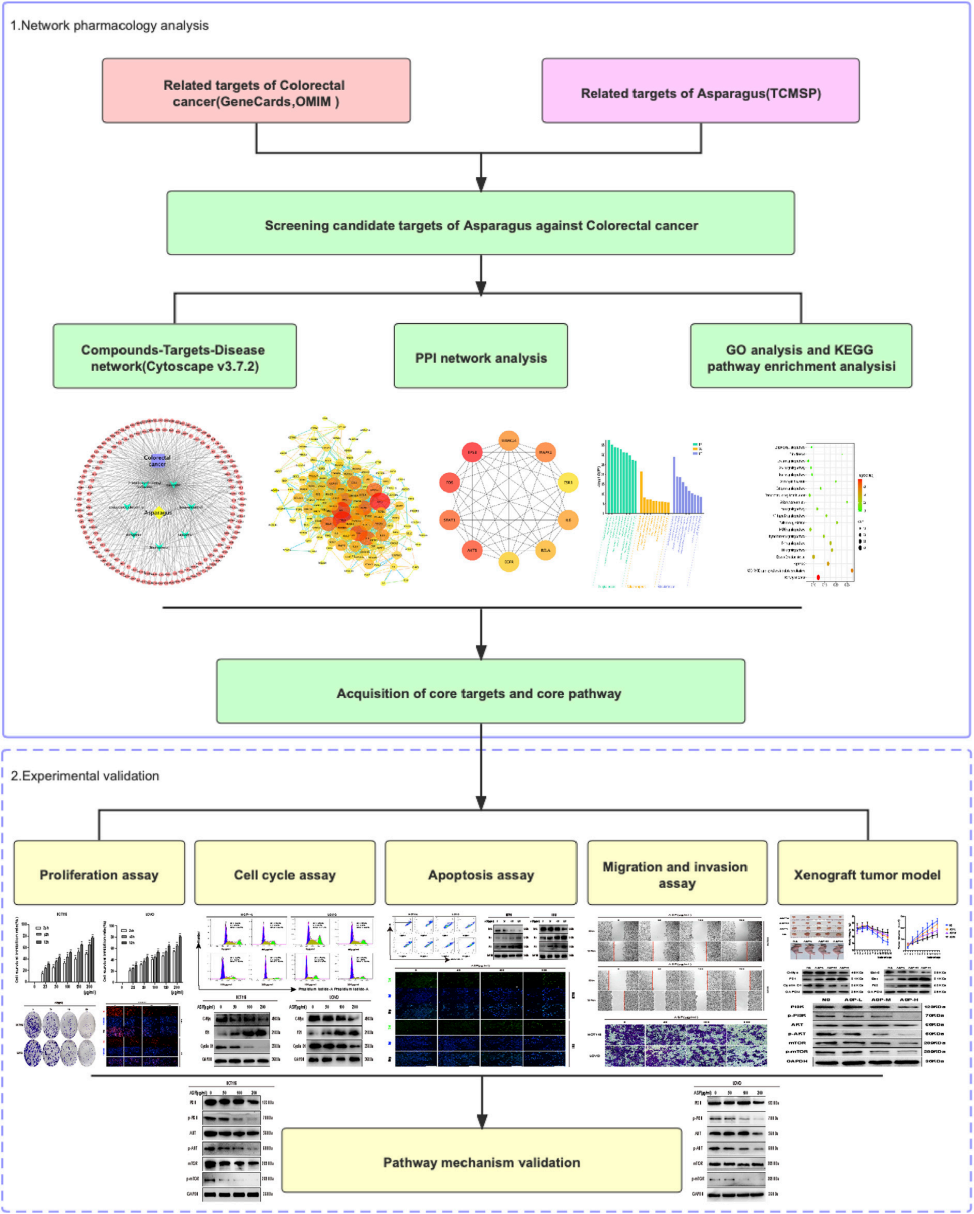
The workflow of the network pharmacology analysis and validation of ASP against CRC.

Network pharmacology is a strategy that combines network science, bioinformatics, and computer science methods into the pharmacological study of TCMs to determine their mechanism of action in the context molecular network regulation. It is holistic and systematic in nature, similar to the holistic view of TCM, and uses the principle of discriminative treatment, providing a more scientific and effective strategy for modern TCM research (Zhang et al., 2019). The aim of the present study was to identify the natural compounds of ASP through network pharmacology, explore the key targets of ASP for the treatment of CRC, understand their potential mechanism of action, and provide a basis for the development and application of ASP. The flow chart of the study is shown in Figure 1.

## 2 Methods and Materials

### 2.1 Network Pharmacology

The systematic Pharmacology Database of Traditional Chinese Medicine (TCMSP,http://tcmspw.com/tcmsp.php) (Zhang et al., 2019) was used to screen active ASP compounds. Oral bioavailability and drug similarity were set to ≥30% and ≥0.18 using a pharmacokinetic information retrieval filter based on the TCMSP platform to obtain qualified herbal compounds. The chemical structures of the corresponding compounds were downloaded using the PubChem database (https://pubchem.ncbi.nlm.nih.gov/). The GeneCard database (https:// www.genecards.org/) and OMIM database (http://www.omim.org/updated in 2020) were used to predict and screen of CRC targets. Oliveros, J. C. Venny 2.1was used to screen for common targets between ASP and CRC-related targets (https://bioinfogp.cnb.csic.es/tools/venny/index.html).

Cytoscape V 3.7.2 (http://www.cytoscape.org/) software was used to construct drug compound-disease-target networks and to analyze core compounds using the merge function. The STRING database platform was used to construct protein-protein (PPI) interaction networks for common ASP and CRC-related targets with a medium confidence level (0.7) and to reject target proteins independent of the network. Metascape (http://metascape.org/) was used for gene ontology (GO) analysis and Kyoto Encyclopedia of Genes and Genomes (KEGG) pathway analysis (Zhou et al., 2019).

### 2.2 Experimental Validation

#### 2.2.1 Preparation of ASP

Pieces of ASP were purchased from GuizhouRen Ji Tang Company Limited (Guiyang, China). Fifty grams of raw drug were crushed, placed in 10 times the volume of purified water, soaked for 2 h, extracted twice at reflux, 1 h/time, filtered, combined filtrate, made up the reduced weight with water, concentrated at 70°C under reduced pressure, and concentrated to 1 g/ml of raw drug. Two ml of the concentrated solution was diluted to 10 ml using purified water, filtered through a 0.22 μm pore size filter, divided into sterile centrifuge tubes, and stored at −20°C.

#### 2.2.2 Cell Culture

Human CRC cell lines (HCT116, LOVO, and LO2) were provided by the Shanghai Institute of Cell Biology, Chinese Academy of Sciences. Cells were cultured in high-sugar Dulbecco’s modified Eagle medium (DMEM, GIBCO, Grand Island, NY, United States) supplemented with 10% fetal bovine serum (FBS, BI, Kibbutz, Israel) and 1% penicillin-streptomycin (Solarbio, Beijing, China). Cells were cultured at 37°C in a 5% CO_2_ atmosphere.

#### 2.2.3 Cell Viability Assay

CRC and LO2 cells100 μl/well (6 × 10^3^ cells/ml) were inoculated into 96-well plates and incubated overnight in a humidified incubator at 37°C and 5% CO_2_. Cells were then pretreated with ASP (0, 25, 50, 100, 150, and 200 μg/ml) for 24, 48, and 72 h. Then, 10 μl of Cell Counting Kit-8 (CCK-8) (Dojindo, Kumamoto, Japan) was added to each well, and the cells were incubated at 37°C, 5% CO_2_ for 4 h. Finally, the absorbance was measured at 450 nm using a Microplate Reader (Thermo Fisher Scientific, Waltham, MA, United States) and the cell proliferation inhibition rate was calculated. Cell proliferation inhibition rate = (1—absorbance value of experimental group/absorbance value of control group) × 100%.

#### 2.2.4 Crystalline Violet Staining

When cell growth reached 70%−90% confluence, cells were digested (Trypsin-EDTA solution, 0.25%with phenol red, Solarbio, Beijing, China) and collected in 6-well cell culture plates at a cell density of 1 × 10^5^ cells/ml. Cells were incubated in a 5% CO_2_ cell culture incubator at 37°C for 24 h. When cells reached 50% confluence, they were exposed to ASP (0, 50, 100, and 200 μg/ml) for 24 h. Cells were fixed with 4% paraformaldehyde, washed twice with phosphate buffered saline (PBS, Solarbio, Beijing, China), and stained with 1% crystalline violet solution (Solarbio). Excess crystal violet dye was washed away using tap water. After natural drying at room temperature, the morphological changes of the cells were observed, analyzed, and photographed under an inverted microscope (Leica DM2500, Heidelberg, Germany).

#### 2.2.5 Colony Formation Assay

Cells were inoculated into 6-well plates at 700 cells per well and treated with different concentrations of ASP for 24 h. Cells were washed with PBS and culture was continued in complete growth medium, which was replaced with fresh medium every 3 days for a total of 14 days. Colonies were fixed with 4% paraformaldehyde and stained with 1% crystalline violet solution for 15 min. The colonies were observed under a light microscope and manually counted in three randomly selected fields to measure the cell colony number (>50 cells/colony).

#### 2.2.6 5-Ethynyl-2’-deoxyuridine (EdU) Assay

CRC cells in logarithmic phase of growth (5,000 cells per well) were inoculated into 96-well plates and incubated for 24 h. Cells were exposed to 0, 50, 100, and 200 μg/ml of ASP and incubation was continued for 24 h. EdU (RiboBio, Guangzhou, China) labeling solution at a dilution of 1,000:1 was added and incubated for 2 h. The remaining operations were completed according to the manufacturer’s instructions, taking care to avoid light. Cells were then imaged under an inverted fluorescence microscope (Nikon, Tokyo, Japan, three random positions in each well were photographed, and the fluorescence of EdU-positive cells was measured using ImageJ 1.8.0 software (NIH, Bethesda, MD, United States). EdU-positive cells (%) = red EdU count/blue Hoechst count 33,342 × 100.

#### 2.2.7 Flow Cytometry to Assay the Cell Cycle

HCT116 and LOVO cells were treated with 0, 50, 100, and 200 μg/ml of ASP for 24 h. The collected cells (1 × 10^6^) were fixed in cold ethanol and stored overnight at 4°C. The next day, cells were washed twice with cold PBS; then 100 μl of RNase A (25 μg/ml) and 400 μl of propidium iodide (PI, 50 μg/ml) were added to each sample according to the instructions of the cell cycle kit (KeyGEN, Nanjing, China) and incubated for 40 min in the dark. The cells were analyzed using a flow cytometer (BD Biosciences, San Jose, CA, United States) and FlowJo 10.0 software (FlowJo, Ashland, OR, United States).

#### 2.2.8 Flow Cytometry to Assay Cell Apoptosis

Cells (1 × 10^5^ cells/ml) were inoculated in 6-well plates and incubated for 24 h and then reacted with the indicated concentrations of ASP for 24 h. Cells were collected according to the instructions of the Fluorescein isothiocyanate (FITC) Annexin V/PI Apoptosis Detection Kit (KeyGEN), and 500 μl of Binding Buffer was added to each sample tube to prepare a cell suspension, the Annexin V-FITC/PI fluorescent dye was added, and incubated for 15 min in the dark. The cells were then measured using flow cytometry (BD Biosciences) and the data were analyzed using FlowJo 10.0 software.

#### 2.2.9 Terminal Deoxynulceotidyl Transferase Nick-End-Labeling (TUNEL) Assay

CRC cells were inoculated at 4,000 cells per well into 96-well plates and incubated for 24 h. The cells were then treated with the indicated concentrations of ASP for 24 h. Cells were fixed in 4% paraformaldehyde for 20 min at room temperature. Apoptosis was detected using the TUNEL Apoptosis Assay Kit (Beyotime, Shanghai, China). TUNEL assay solution (50 μl) was added to each well according to the manufacturer’s protocol and incubated for 60 min at 37°C in the dark. The cell nuclei were counterstained using 4’,6-diamidino-2-phenylindole (DAPI). TUNEL-positive cells were imaged under a fluorescence microscope and measured using the ImageJ 1.8.0 software. TUNEL-positive cells (%) = Green TUNEL count/blue DAPI count × 100.

#### 2.2.10 Wound Healing Assay

Cells (2×10^5^ cells/well) were inoculated onto 6-well plates and incubated for 24 h in a 37°C incubator. A sterile pipette tip was used to make a scratch in the cell monolayer, the cells were washed three times with PBS to dislodge the cells debris, photographed under an inverted microscope, and the culture was continued with fresh medium without FBS in the presence of the indicated concentrations of ASP for 24 h. Six different locations were photographed randomly under an inverted microscope, and the wound area was analyzed using ImageJ 1.8.0 software. Wound healing was assessed according to the size of the wound at 0 h and 24 h.

#### 2.2.11 Transwell Invasion Assay

Transwell invasion assays were performed using an 8 µm well Transwell chamber system (Corning Inc. Corning, NY, United States). The upper chamber was coated with Matrigel matrix gel (BD Biosciences). Cells (5 × 10^4^ cells/well) after 24 h of treatment were resuspended in 200 µl of FBS-free medium, inoculated into the upper chamber, and 600 µl of complete medium containing 10% FBS was added to the lower chamber. After 24 h of incubation, uninvaded cells were removed from the upper surface of the membrane using a cotton swab, and the invaded cells were fixed and stained with 1% crystal violet solution. Finally, light microscopy was performed to photograph and count six random fields of view in each group.

#### 2.2.12 Western Blotting Analysis

Total cellular or tissue proteins were extracted using Radioimmunoprecipitation assay (RIPA) lysis buffer containing protease inhibitor, phosphatase inhibitor, and PMSF (Solarbio, Beijing, China). Equal amounts of proteins were subjected to 10% sodium dodecyl sulfate polyacrylamide gel electrophoresis (SDS-PAGE) (Solarbio) for separation and then transferred to polyvinylidene difluoride (PVDF) membranes (0.45 µm, Millipore, Unites States). The membranes were blocked using with 5% skim milk for 1 h at room temperature, and then incubated with primary antibodies overnight at 4°C. The next day, the membranes were washed three times with Tris-buffered saline-Tween 20 (TBST, Solarbio) and incubated with goat anti-rabbit or goat anti-mouse horseradish peroxidase (HRP)-coupled secondary antibodies for 2 h at room temperature. Finally, immunoreactive protein bands were visualized using ultrasensitive ECL chemiluminescent ready-to-use substrate (BOSTER, Wuhan, China). The grayscale values of the protein bands were quantified using ImageJ software and the relative level of the proteins was normalized by the level of glyceraldehyde-3-phosphate (GAPDH). The antibodies used were as follows: anti-tumor protein 53 (TP53), anti- BCL2 associated X (Bax), anti-BCL2 apoptosis regulator (Bcl-2), anti-Cyclin D1, anti-P21, anti-C-Myc, anti-PI3K, anti-phospho (p)-PI3K, anti-protein kinase B (AKT), anti-p-AKT, anti-mechanistic target of rapamycin kinase (mTOR), anti-p-mTOR, and anti-GAPDH 1:1,000 (Beyotime), goat anti-rabbit and goat anti-mouse HRP-coupled secondary antibodies 1:5,000 (BOSTER).

### 2.3 Nude Mice Tumor Xenografts

Twenty male BALB/c nude mice (4 weeks old, 20 ± 2 g) were purchased from Spelford Biotechnology Ltd. (Beijing, China). All animal experiments were performed in the pathogen-free medical animal laboratory of theFirst Affiliated Hospital of Guizhou University of Traditional Chinese and were approved by the Animal Ethics Committee of the First Affiliated Hospital of Guizhou University of Traditional Chinese (approval number: AHQU20180310A). After 3 days of acclimatization in a specific pathogen free (SPF)-grade animal room, 200 μl (5 × 107 cells/ml) of HCT116 cells were implanted subcutaneously into the right axilla of each nude mouse. At 7 days after cell inoculation, non-tumor-forming and tumor-oversized mice were excluded, and then the mice were randomly divided into four groups: control (*n* = 5, 0.9% saline, 0.1 ml/10 g) and ASP-L, ASP-M, and ASP-H groups (*n* = 5, ASP at 100, 200, and 300 mg/kg, 0.1 ml/10 g, respectively). The drug concentration in the ASP-treated group was derived from a previous study (Agrawal et al., 2008). The drug was administered via gavage, once daily for 2 weeks. The body weight and tumor size of nude mice were measured every other day. The tumor volume was determined by measuring the length (L) and width (W) and calculated according to the following formula: tumor volume (mm3) = 0.5 × L × W2). At the end of the experiment, the neck of nude mice was dislocated, and the tumors were excised, weighed, and photographed.

### 2.4 Statistical Analysis

Data analysis was performed using GraphPad Prism 8.3.1 software (San Diego, CA, United States). All experimental data are expressed as the mean ± SD. The statistical significance of the results was analyzed using one-way analysis of variance (ANOVA) for multiple group comparisons and by Student’s t-test for two group comparisons. A *p* value < 0.05 indicates a significant difference. All experiments were performed in triplicate.

## 3 Results

### 3.1 Network Pharmacological Analysis of ASP Targeting CRC

In this study, using the TCMSP database (Table 1), we retrieved a total of nine compounds (among which methylprotodioscin_qt and Asparaside A_qt had no relevant targets) from ASP using Oral Bioavailability (OB) ≥30% and Drug-like (DL) ≥0.18 as screening conditions.

**TABLE 1:** Active ingredients andabsorbed, distributed, metabolized and excreted (ADME) parameters of ASP.

Mol ID	Molecule name	Structure	OB (%)	DL
MOL000098	quercetin	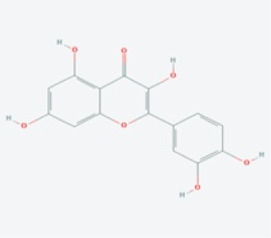	46.43	0.28
MOL000358	beta-sitosterol	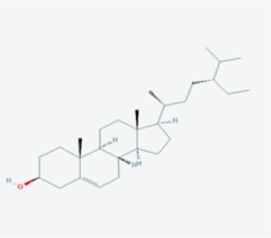	36.91	0.75
MOL000359	sitosterol	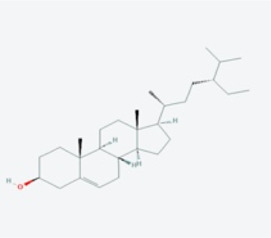	36.91	0.75
MOL000449	Stigmasterol	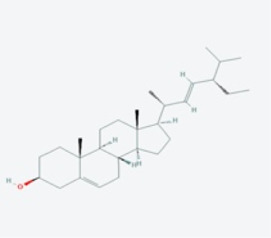	43.83	0.76
MOL000546	diosgenin	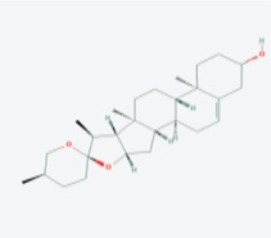	80.88	0.81
MOL003889	methylprotodioscin_qt	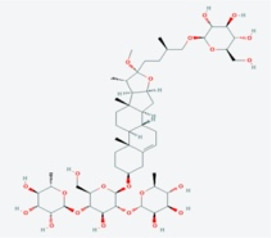	35.12	0.86
MOL003891	pseudoprotodioscin_qt	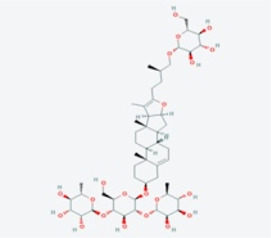	37.93	0.87
MOL003896	7-Methoxy-2-methyl isoflavone	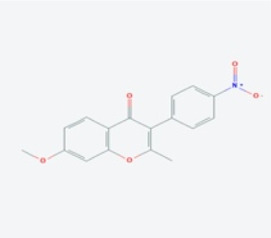	42.56	0.20
MOL003901	Asparaside A_qt	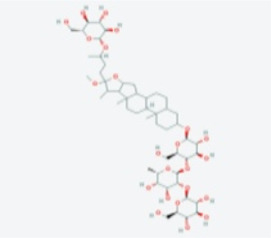	30.60	0.86

**FIGURE 2:**
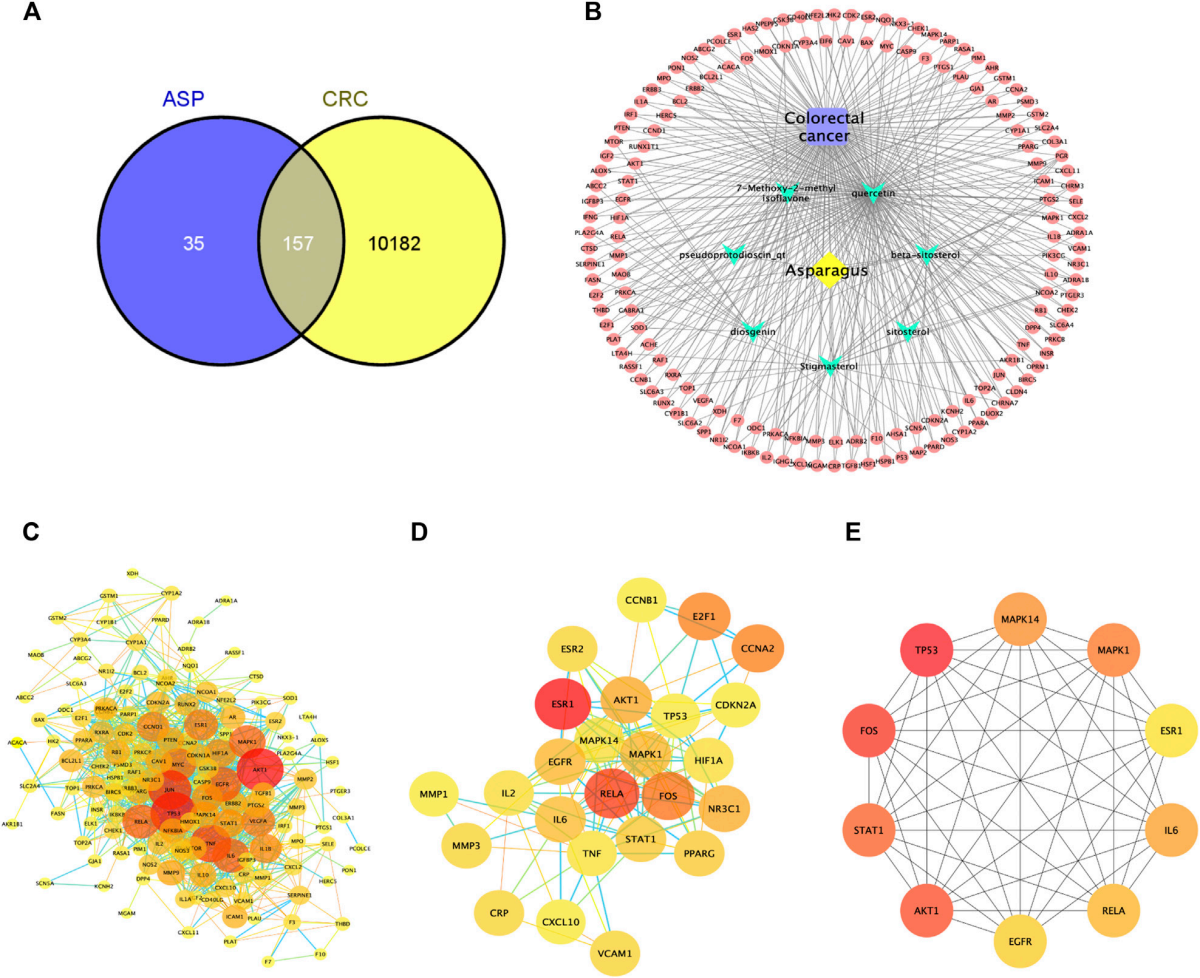
Network pharmacological analysis of asparagus (ASP). **(A)** Venn diagram showing the 157 common targets of ASP in colorectal cancer (CRC). **(B)** Compound-disease-target network of ASP against CRC. **(C)** Protein-protein interactions identified by STRING software (visualized by Cytoscape 3.7.1 software). **(D)** Cluster subnetwork based on MCODE analysis with a score of 10.667. **(E)** Top 10 core nodes derived according to the MCC algorithm.

From these 7 compounds, 192 relevant genes were screened. 10,339 genes associated with CRC were screened in GeneCard and OMIM databases (duplicates were removed). A total of 157 common targets were obtained using Venny 2.1.0 (Figure 2A), and these intersections were considered as potential candidate targets for ASP against CRC. Seven active ingredients and 157 “drug-disease”" key target genes were introduced into Cytoscape 3.7.1 software. A visualized active ingredient-cancer target network was constructed (Figure 2B). The active ingredients with the most target genes in ASP were quercetin, beta-sitosterol, and 7-Methoxy-2-methyl isoflavone.

The target PPI network was constructed using the STRING database platform, and 157 target genes were introduced. The selected species was *Homo sapiens*, and the PPI network with confidence level >0.7 and potential targets of ASP against CRC is shown in Figure 2C. The PPI network consists of 141 hub nodes and 972 edges (after removing the free nodes). The results show that there were five cluster subnetworks based on MCODE analysis, where the highest scoring cluster subnetwork made up of 25 hub nodes and 128 edges. The clusters included: RELA, STAT1, ESR1, AKT1, MAPK1, IL2, MAPK14, CCNB1, CRP, TP53, CCNA2, PPARG, VCAM1, CXCL10, FOS, ESR2, E2F1, MMP3, IL6, TNF, EGFR, CDKN2A, MMP1, HIF1A, and NR3C1, with a score of 10.667 (Figure 2D). Moreover, the top 10 core nodes were obtained in the order of the MCC algorithm score from highest to lowest, namely TP53, FOS, AKT1, STAT1, MAPK1, MAPK14, IL6, RELA, EGFR, and ESR1 (Figure 2E). These results suggest that these core targets may contribute to the fundamental therapeutic role of ASP in CRC.

**FIGURE 3:**
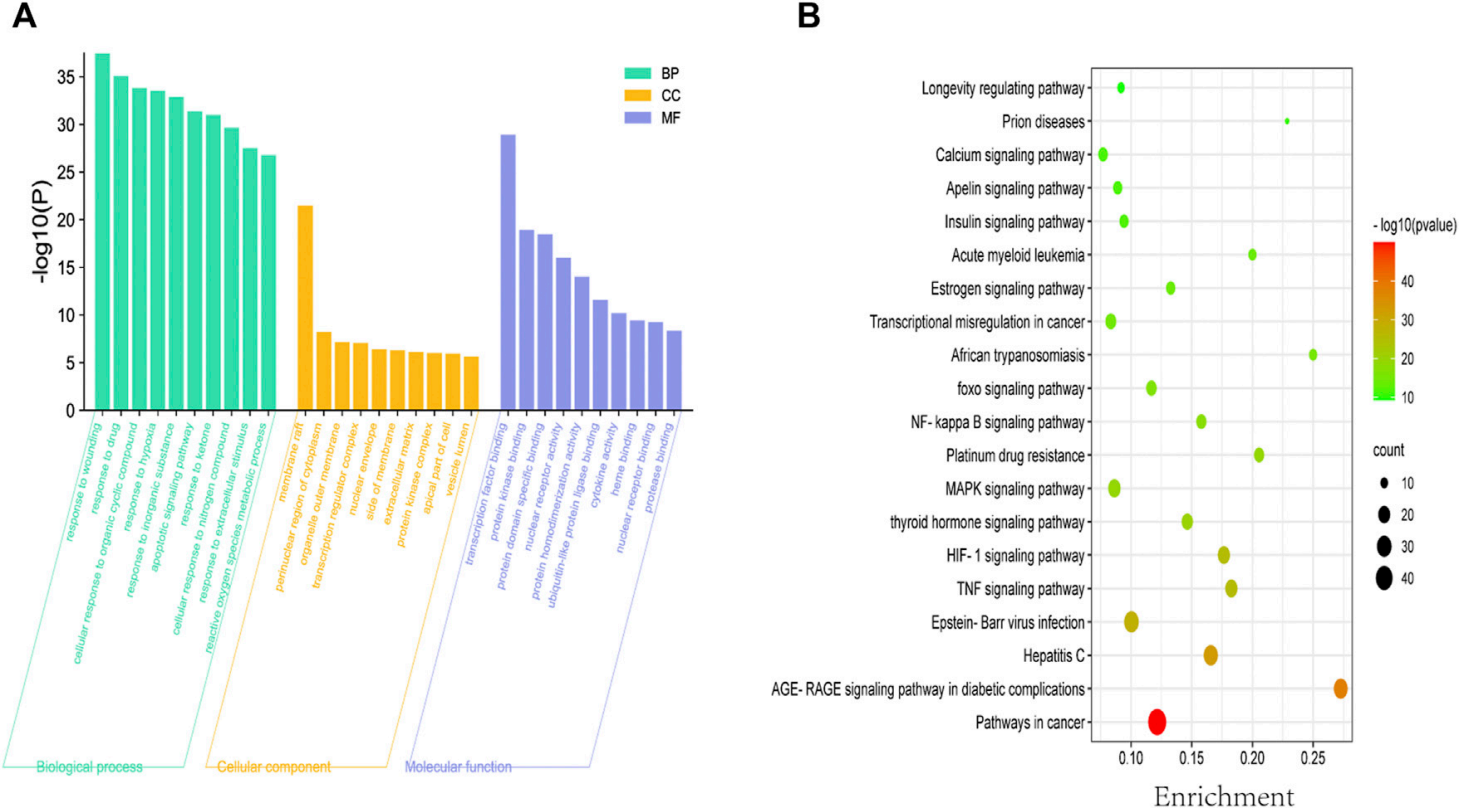
Biofunctional enrichment analysis of *Asparagus* (ASP). **(A)** Top 10 biological processes, top 10 molecular functions and top 10 cellular components in the GO analysis. **(B)** Top 20 KEGG pathways. The color scale indicates the different thresholds of *p* values and the size of the dots represents the number of genes corresponding to each term.

To explore the therapeutic mechanisms of putative ASP targets for CRC, GO and KEGG pathway enrichment analysis was performed using Metascape (updated 2021-02-01). A total of 2068 biological processes (BP), 89 cellular components (CC) and 165 molecular functions (MF), respectively, satisfied the requirements of gene count ≥3 and *p*-value ≤ 0.01. The top 10 significantly enriched GO terms in BP, CC and MF are plotted in Figure 3A, and the results showed that ASP’s effects might be mediated through response to wounding, response to drug, membrane raft, perinuclear region of cytoplasm, organelle outer membrane, transcription factor binding, protein kinase binding, and protein domain specific binding, to regulate the proliferation and apoptosis of CRC cancer cells, thus exerting its anti-cancer effects on CRC. A total of 329 related signaling pathways were obtained by KEGG enrichment analysis. The top 20 signaling pathways with high confidence and *p*-values ≤ 0.01 were selected for analysis, as shown in Figure 3B. From Figure 3B, it can be seen that most of the target genes affect substances or signaling pathways, including Pathways in cancer, AGE-RAGE signaling pathway in diabetic complications, Hepatitis C, Epstein-Barr virus infection, and TNF signaling pathway. We found that Pathways in cancer play an important role in ASP against CRC. This pathway is large and complex, including ERK signaling, PI3K Signaling, WNT Signaling, NOTCH Signaling, Other RAS Signaling, etc.

### 3.2 ASP Inhibited the Proliferation of CRC Cells *in vitro*

**FIGURE 4:**
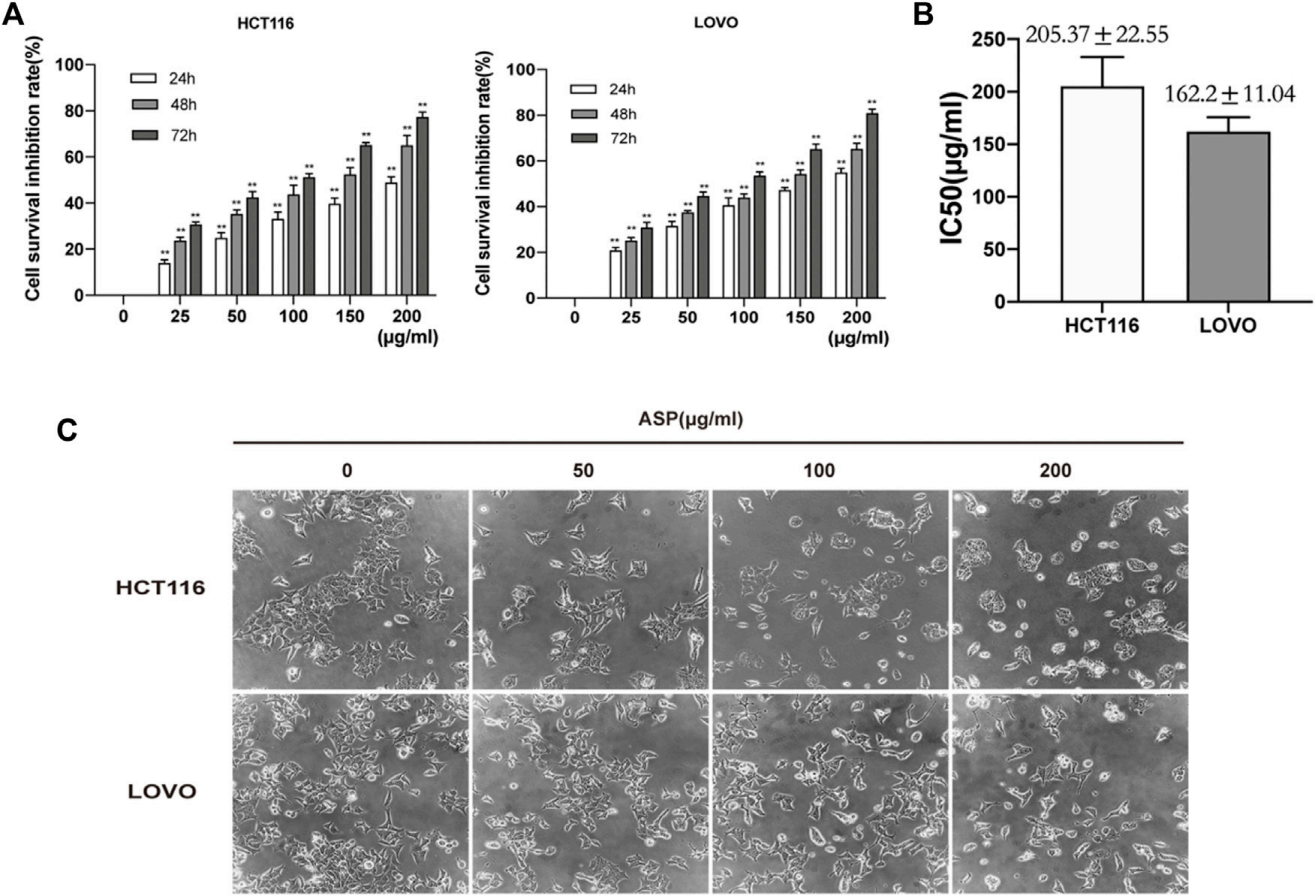
*Asparagus* aqueous extract (ASP) inhibits the proliferation of HCT116 and LOVO cells. **(A)** Cell Counting Kit-8 (CCK-8) analysis of human colorectal cancer (CRC) cell lines (HCT116 and LOVO cells) after treatment with the indicated doses of ASP for 24, 48, and 72 h. **(B)** Median inhibitory concentrations (IC50) of ASP acting against HCT116 and LOVO cells for 24 h. **(C)** Morphological changes of cells observed by light microscopy after 24 h of ASP application at the indicated concentrations. *p < 0.05, **p < 0.01 *vs*. control.

CRC cells (HCT116, LOVO, and LO2) were pretreated with different concentrations of ASP (0−200 μg/ml) for 24, 48, and 72 h. CCK-8 analysis showed that ASP significantly inhibited the viability of CRC cells in a dose- and time-dependent manner (Figure 4A). However, the cytotoxicity of ASP to normal LO2 cells was significantly lower than that of CRC cells (Supplementary Figures S1A,B). The median inhibitory concentration (IC50) values for HCT116 and LOVO cells were 205.37 ± 22.55 μg/ml and 162.2 ± 11.04 μg/ml for 24 h (Figure 4B). Light microscopy images showed a gradual decrease in the number of cells with increasing ASP concentration, poor cell wall adhesion, cell wrinkling, varying degrees of nuclear consolidation, and nuclear vacuolization (Figure 4C). These data suggest that ASP has cytotoxic effects.

**FIGURE 5:**
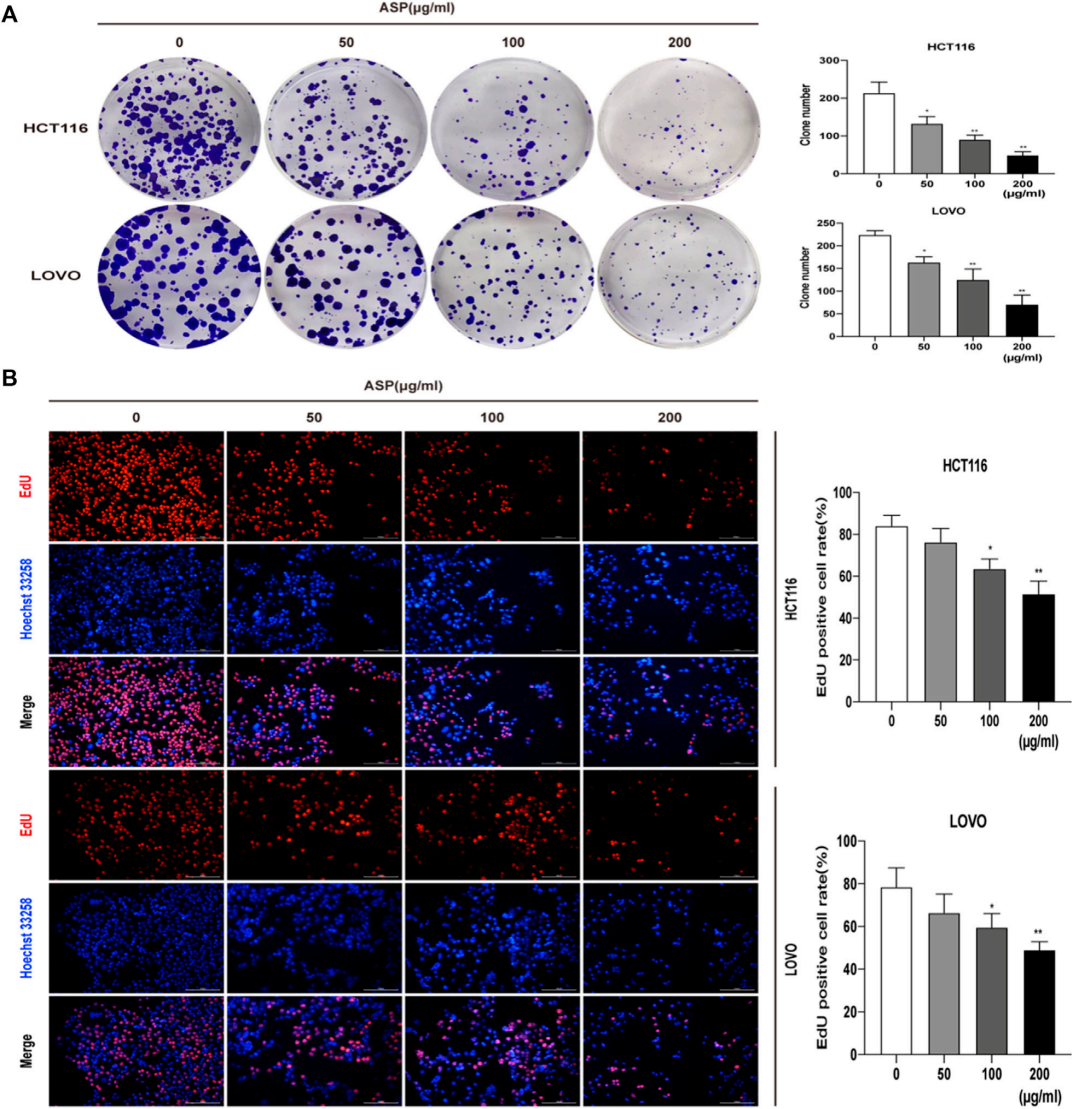
**(A)** Colony formation assays were performed on HCT116 and LOVO cells cultured with different doses (0, 50, 100. and 200 μg/ml) of ASP. The number of colonies formed was calculated and expressed as mean ± standard deviation (SD) (*n* = 3). **(B)**The proliferation capacity of HCT116 and LOVO cells treated with the indicated concentrations of ASP (0, 50, 100, and 200 μg/ml) for 24 h was measured using an EdU assay (×200). *p < 0.05, **p < 0.01 *vs*. the control.

We used colony formation and EdU assays to investigate the effect of ASP on the proliferation of CRC cells (HCT116 and LOVO). As shown in Figure 5A, ASP inhibited the colony formation of HCT116 and LOVO cells at the indicated concentrations. The EdU assay further showed that ASP reduced the percentage of EdU-positive cells in a dose-dependent manner (Figure 5B). These findings clearly indicate that ASP has an anti-proliferative effect on HCT116 and LOVO cells.

**FIGURE 6:**
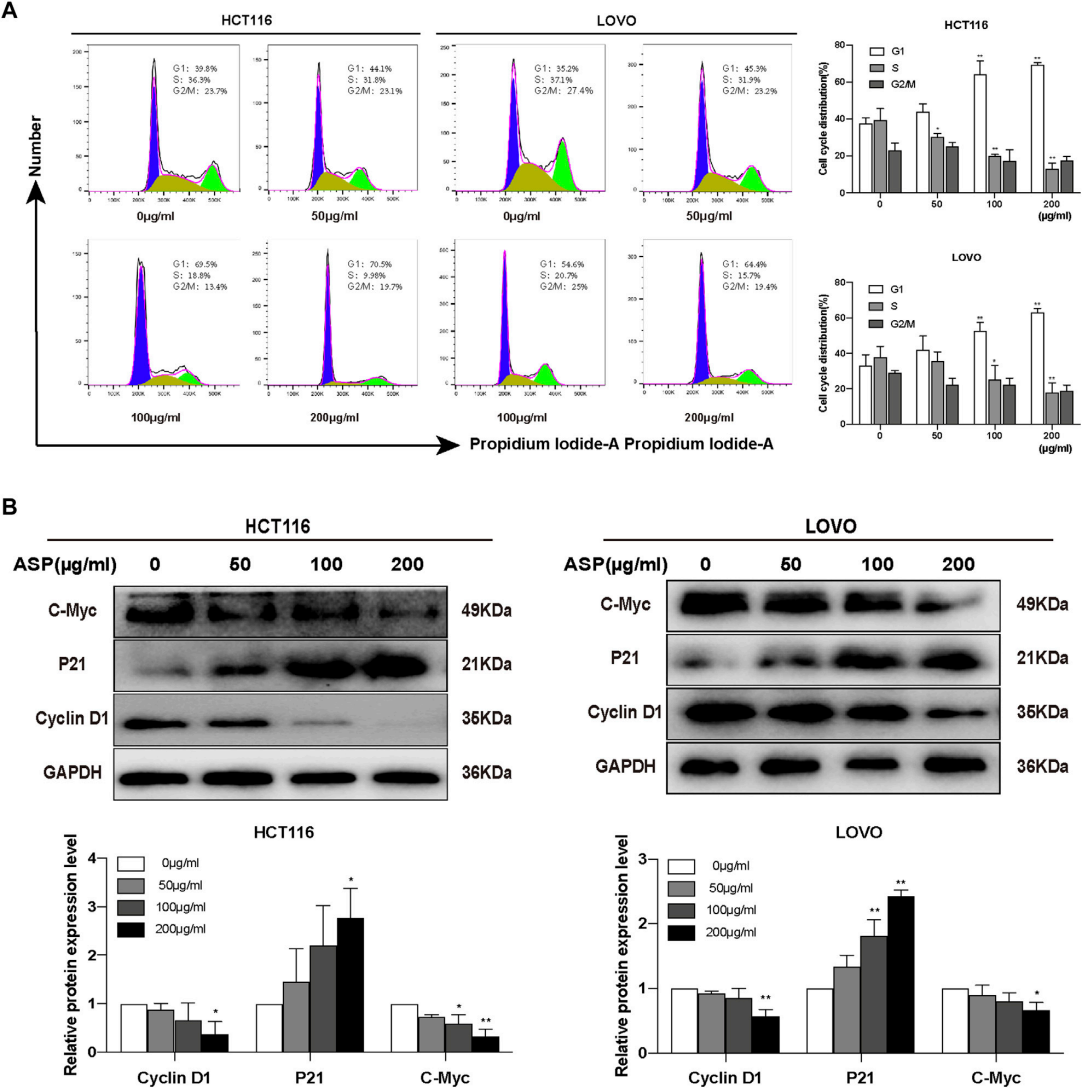
*Asparagus* aqueous extract (ASP) significantly inhibits the cell cycle of colorectal cancer cells. **(A)** ASP significantly inhibited the cell cycle progression of HCT116 and LOVO, stopping them in G1/G0 phase, as shown in flow cytometric analysis (*n* = 3 per group). **(B)** Expression of Cyclin D1 and C-Myc decreased with increasing ASP concentration and P21 increased (*n* = 3 per group). Values are shown as the mean ± SD, *p < 0.05, **p < 0.01 *vs*. the control.

Cell cycle analysis showed that the percentage of HCT116 cells in the S phase decreased significantly with increasing ASP concentrations compared with controls, whereas the number of cells in the G0/G1 phase increased significantly (Figure 6A). Similar results were obtained in LOVO cells (Figure 6A). ASP inhibited the expression of Cyclin D1 (200 μg/ml *p* < 0.05) and C-Myc (100 and 200 μg/ml *p* < 0.05) proteins, and promoted the expression of P21 in HCT116 cells (200 μg/ml *p* < 0.05, Figure 6B); in LOVO cells, similar results were observed. These data further suggested that ASP has an inhibitory effect on cell proliferation.

### 3.3 ASP Promoted CRC Cell Apoptosis *in vitro*

**FIGURE 7:**
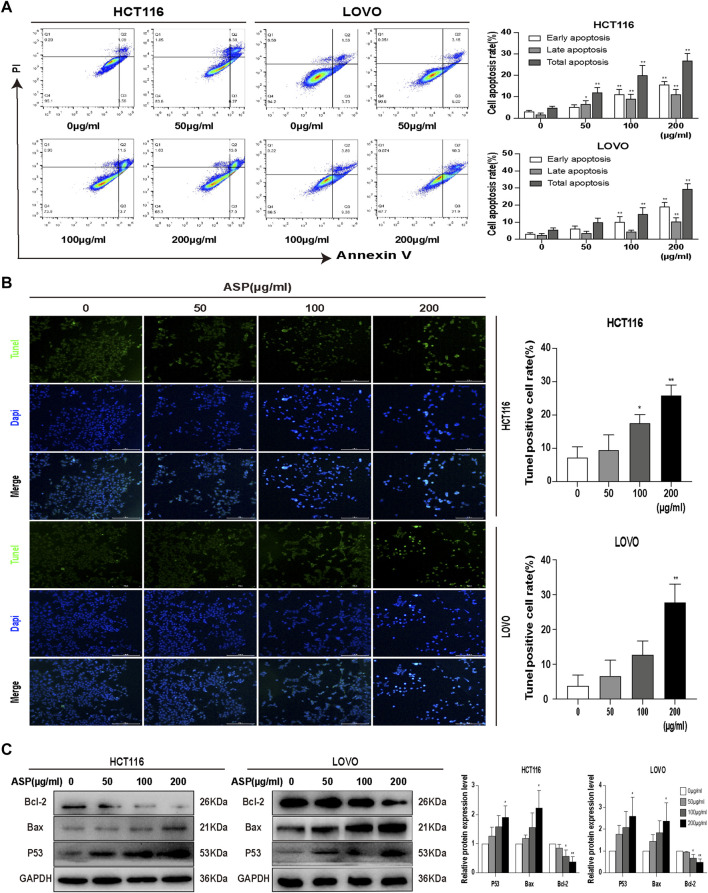
*Asparagus* aqueous extract (ASP) significantly promoted apoptosis in colorectal cancer cells. **(A)** Flow cytometry analysis showed that ASP promoted early and late apoptosis in HCT116 and LOVO cells (n = 3 in each group). **(B)** TUNEL staining analysis showed that ASP promoted apoptosis (×200). **(C)** Expression of Bcl-2 decreased and expression of TP53 and Bax increased with increasing ASP concentration (*n* = 3 per group) Values are shown as the mean ± SD, *p < 0.05, **p < 0.01 *vs*. the control.

Flow cytometry was used to detect the effect of different concentrations of ASP on the apoptosis of CRC cells. The results showed that when treated with 0, 50, 100, and 200 μg/ml of ASP, the total apoptosis rate of HCT116 cells increased with increasing ASP concentrations, with early (100 and 200 μg/ml *p* < 0.01) and late (50, 100, and 200 μg/ml *p* < 0.05) apoptosis being significantly promoted; similar results were obtained for LOVO cells (Figure 7A). Similarly, TUNEL staining showed an increase in the percentage of apoptosis after ASP treatment (Figure 7B). In HCT116 cells, ASP inhibited the expression of Bcl-2 (100 and 200 μg/ml *p* < 0.05) and promoted the expression of TP53 (200 μg/ml *p* < 0.05) and Bax (200 μg/ml *p* < 0.05) proteins; similar results were observed in LOVO cells (Figure 7C). These results suggested that ASP has a pro-apoptotic effect on CRC cells.

### 3.4 ASP Inhibited the Migration and Invasion of CRC Cells *in vitro*

**FIGURE 8:**
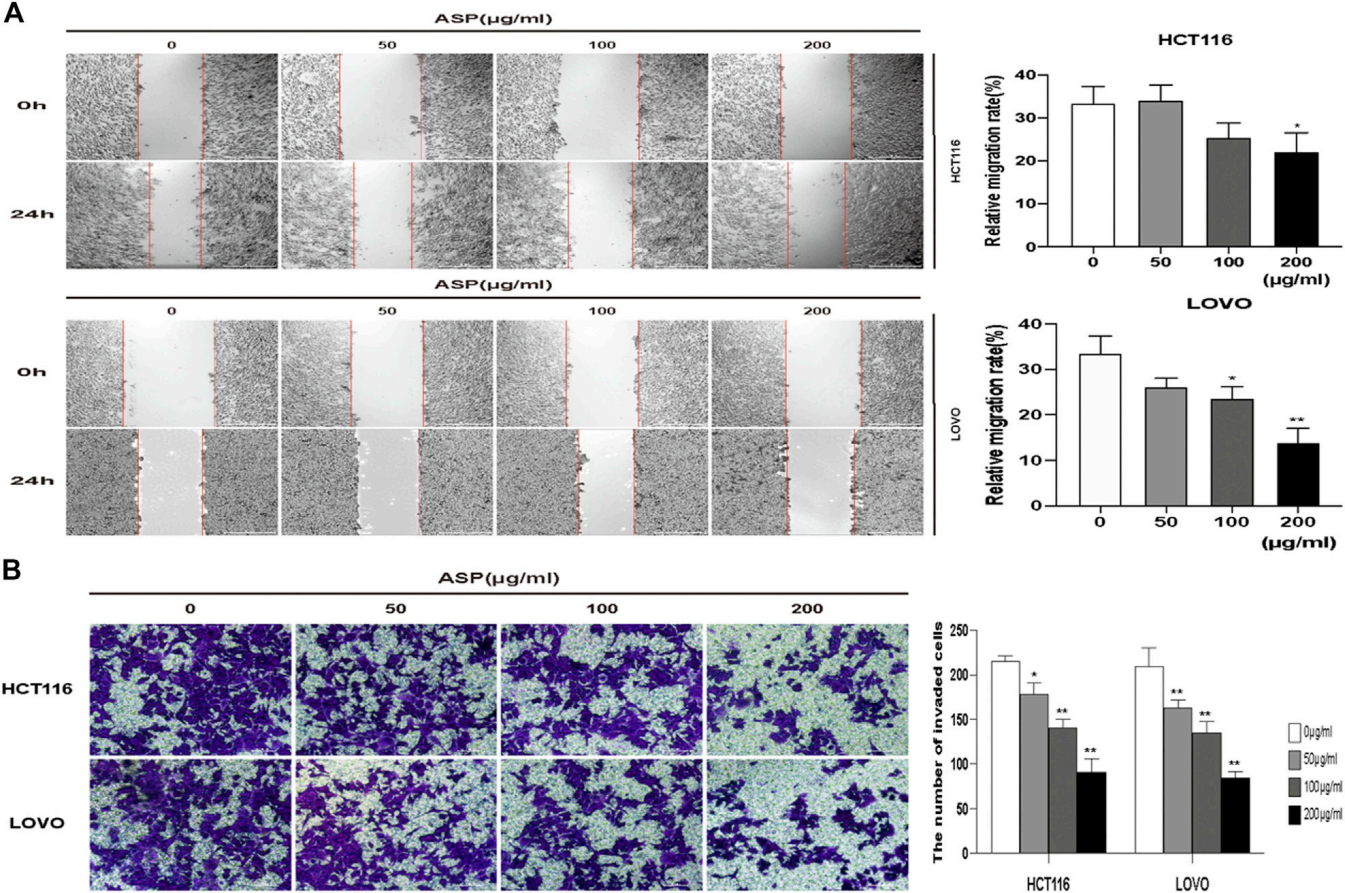
ASP inhibits the migration and invasion of human CRC cells **(A)** Wound healing migration assay of HCT116 and LOVO cells under ASP treatment (×40), *n* = 5. **(B)** Transwell invasion assay of HCT116 and LOVO cells under ASP treatment (×100), n = 6. *p < 0.05, **p < 0.01 *vs*. the control.

We performed wound healing and Transwell invasion assays to explore the effect of ASP on the migration and invasive ability of human CRC cells. As shown in Figure 8A, the migration of HCT116 and LOVO cells was significantly inhibited in the ASP treated group compared with that in the control group. As shown in Figure 8B, in the Transwell invasion assay, invasion of HCT116 and LOVO cells was inhibited in a dose-dependent manner in the ASP-treated group. These results confirmed that ASP inhibits the migration and invasion of CRC cells.

### 3.5 ASP Induced Apoptosis in CRC Cells via the PI3K-Akt-mTOR Pathway

Based on previous informatics data, as shown in Figures 2E, 3B, *AKT1* was one of the key targets in the top 10 bioenrichments, and Pathways in cancer was the most enriched signaling pathway; therefore, wedecided to test whether mTOR/PI3K/AKT pathway involved in the anti-tumor effect of ASP.

**FIGURE 9:**
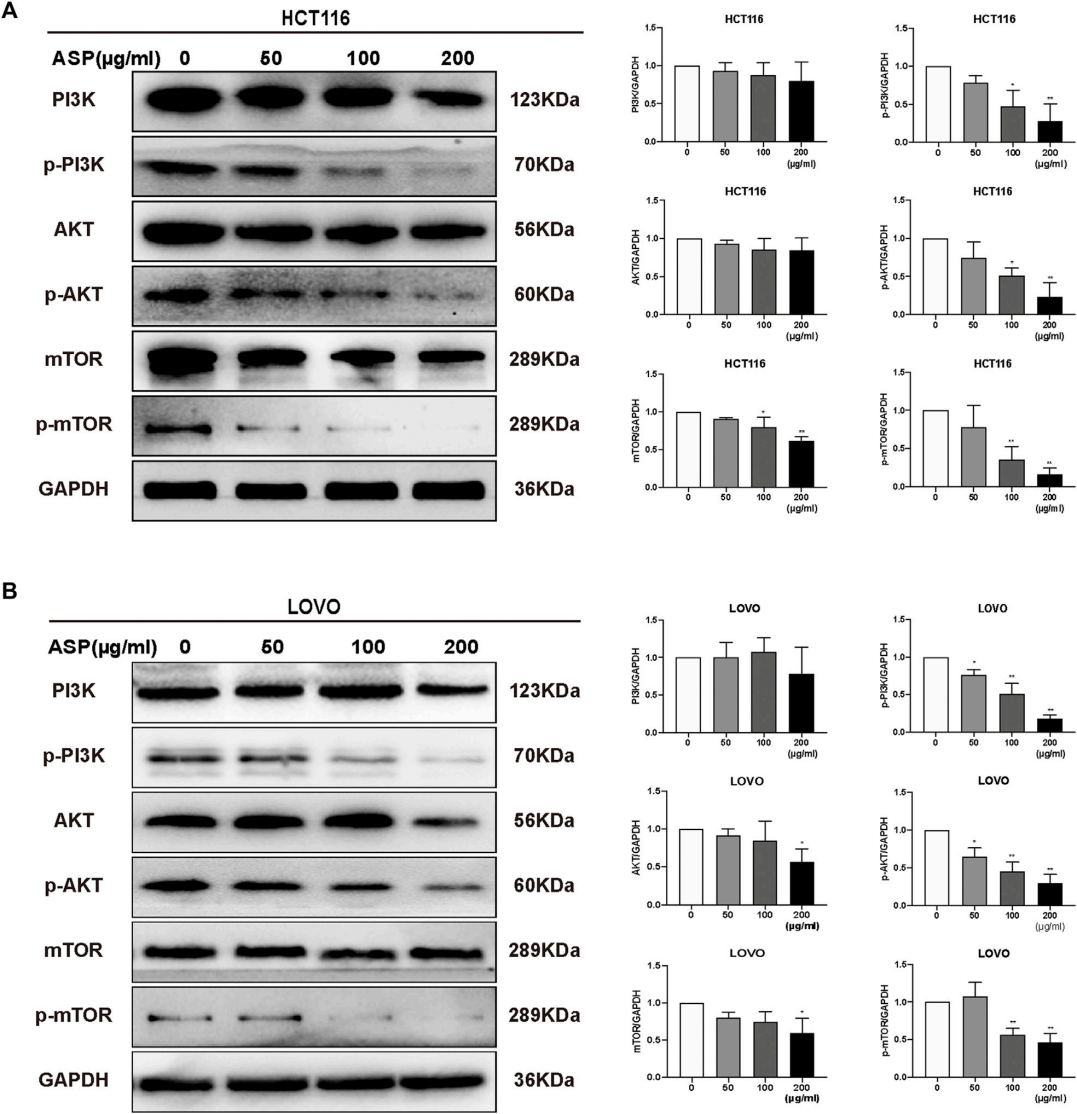
ASP regulates PI3K/AKT/mTOR signaling pathway to induce apoptosis in CRC cells. In HCT116 **(A)** and LOVO **(B)** cells, the levels of PI3K/AKT/mTOR signaling pathway-related proteins were altered by ASP treatment (*n* = 3 in each group). Values are shown as the mean ± SD, *p < 0.05, **p < 0.01 *vs*. the control.

We further investigated the mechanism of how ASP promotes apoptosis using western blotting of HCT116 and LOVO cells. As shown in Figure 9, ASP downregulated the levels of p-PI3K, p-AKT, and p-mTOR (100 and 200 μg/ml *p* < 0.05) proteins in HCT116 and LOVO cells compared with those in the controls. In conclusion, these data support the hypothesis that ASP induces apoptosis in CRC cells by regulating the PI3K/AKT/mTOR pathway.

### 3.6 ASP Inhibited the Growth of HCT116 Cell Xenografts in Nude Mice

**FIGURE 10:**
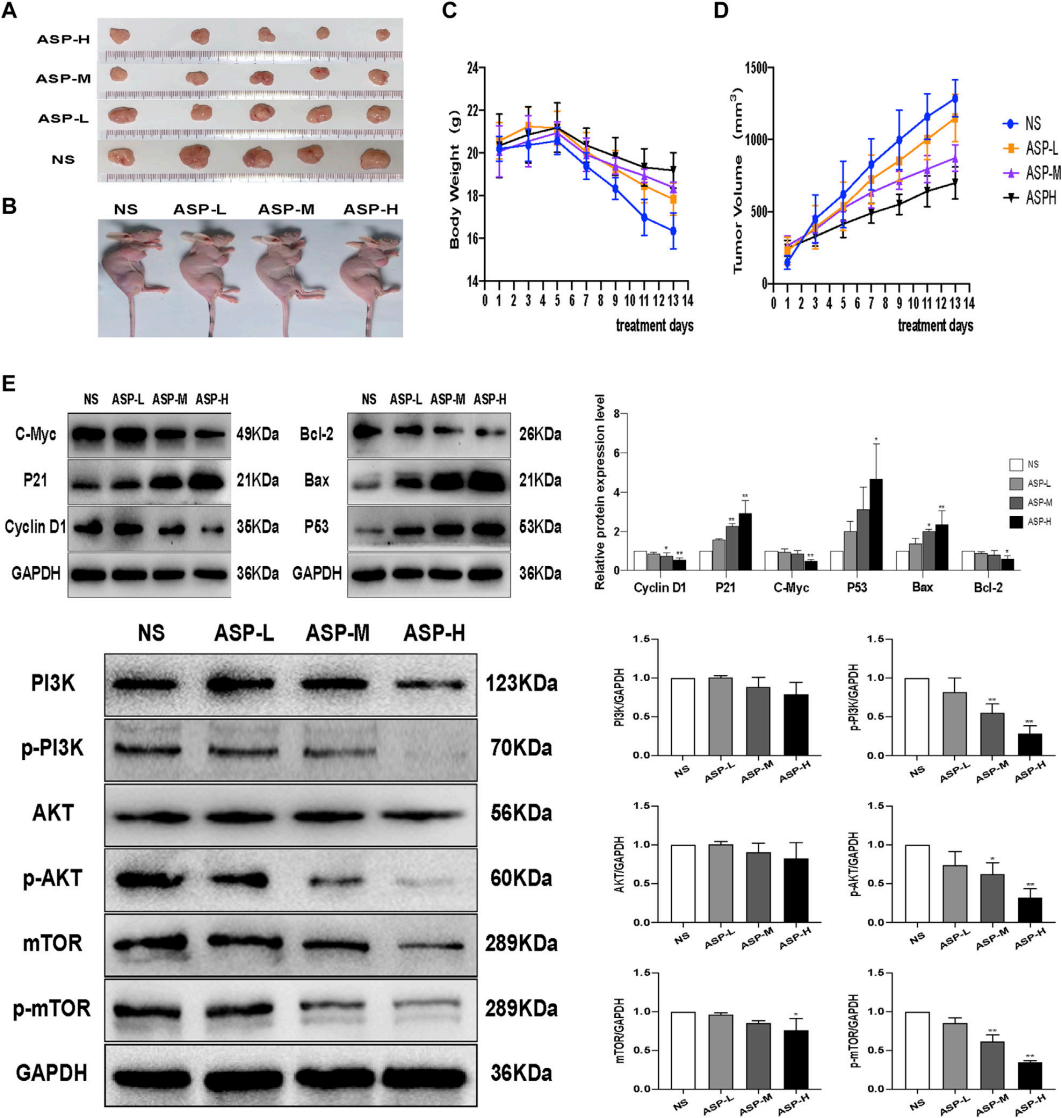
ASP inhibits CRC tumor growth *in vivo*.**(A,B)** Subcutaneous xenograft tumor profiles after 2 weeks (n = 5). **(C)** Changes in body weight of mice during ASP treatment (*n* = 5). **(D)** Changes in tumor volume in mice during ASP treatment (*n* = 5).**(E)** Protein levels of Cyclin D1, P21, C-Myc, TP53, Bcl-2, Bax, PI3K, p-PI3K, AKT, p-Akt, mTOR, and p-mTOR in tumor tissues (*n* = 3). Values are displayed as the mean ± SD, *p < 0.05 and **p < 0.01 *vs*. the control.

The HCT116 cell xenograft tumor model was used to study the antitumor effects of ASP. The results showed that ASP significantly inhibited tumor growth compared to the NS control group (Figures 10A,B). In addition, the rate of weight loss of mice in the ASP-treated group slowed down with increasing drug concentration in the middle and late stages compared with that in the NS group (Figure 10C).At the same time, the rate of tumor volume increase slowed down in the ASP group, although not significantly (Figure 10D). Meanwhile, western blotting analysis of tumor tissues was performed (Figure 10E). Compared with the control group, the levels of p-PI3K, p-AKT, and p-mTOR in mice treated with ASP decreased. Levels of apoptosis markers, including P53 and Bax, were increased and those of Bcl-2 decreased in tumor cells. The levels of cycle-related proteins was consistent with the results of *in vitro* studies. These data suggested that ASP inhibits tumor growth, induces cell cycle arrest and apoptosis in HCT116 cells, and inhibits the PI3K/AKT/mTOR signaling pathway *in vivo*.

## 4 Discussion

Chinese medicine is a great repository of knowledge related to disease prevention and treatment; however, because of the complexity and diversity of chemical components in Chinese medicine, it is difficult to explore the active ingredients and the corresponding mechanism of efficacy after entering the human body, which is the main challenge to research on Chinese medicine. In recent years, network pharmacology technology has been rapidly developed. Its theoretical ideas are similar to the multi-component, multi-target, and multi-pathway action characteristics of TCM, providing new ideas for studying complex TCM systems (Li, 2016).

The anticancer activity of polysaccharide, saponin, and flavonoid extracts from ASP has been reported in various cancers, such as lung and bladder cancer (Bilušić et al., 2019), liver cancer (Cheng et al., 2021), and ovarian cancer (Xu et al., 2021). Some studies have found that asparagus saponin extracts have significant cytotoxic effects on colorectal cancer cells (Jaramillo-Carmona et al., 2018), and the results of Bousserouel (Bousserouel et al., 2013) and others have shown in more detail that methanolic extracts of ASP can induce apoptosis by activating the TRAIL death receptor pathway in SW480 and SW620 human colon adenocarcinoma cells. However, the anticancer effects and mechanisms of ASP on colorectal cancer have not been determined systematically. Therefore, we used a network pharmacology approach to construct a relational network of asparagus active ingredients targeting disease pathways. By analyzing the importance of each node in the topological network, we initially screened the key active ingredients of asparagus against colorectal cancer, including quercetin, β-sitosterol, and 7-methoxy-2-methylisoflavone. Quercetin has been shown to inhibit a variety of cancers, such as nasopharyngeal, colorectal, and breast cancers, by affecting different cancer pathways (Almatroodi et al., 2021). β-sitosterol is known to have a wide range of antitumor effects, and Wang et al. (Wang Z. et al., 2020) found that β-sitosterol can mediate the p53/NF-κB/BCRP signaling axis to modulate the response of CRC to chemotherapy. Jiang et al. (2016) showed a negative correlation between isoflavone intake and colorectal cancer risk in a case-control study. All these studies and our experimental studies support the theory of “different treatment for the same disease” and “different treatment for different diseases” in TCM and demonstrate the good practice of the network pharmacology approach in identifying the mechanism of action of TCM.

We identified 157 potential drug targets, and through PPI network analysis, we identified the core targets among them as TP53, FOS, AKT1, STAT1, MAPK1, MAPK14, IL6, RELA, EGFR, and ESR1. In addition, analysis at the Metascape database (Zhou et al., 2019) showed that multiple signaling pathways are closely related to ASP treatment of CRC, with the highest correlation being “Pathways in cancer,” and combined with the core targets, we speculated that ASP might act therapeutically through the PI3K/AKT/mTOR signaling pathway.

The results of previous studies confirmed that a saponin extract and methanol extract of asparagus have some toxic effects on CRC cells (Bousserouel et al., 2013; Jaramillo-Carmona et al., 2018). Similarly, the results of the present study showed that an aqueous extract of asparagus significantly decreased the proliferation of HCT116 and LOVO human colorectal cancer cells. The same results were observed in xenograft tumor models. The tumor growth rate was reduced in the ASP-treated group compared with that in the NS group. A previous study showed that methanolic extract of asparagus increased the percentage of G2/M phase and apoptosis in MCF-7 cells (Mfengwana et al., 2019). In comparison, our results showed that ASP induced cell cycle arrest in the G0/G1 phase of CRC cells and similarly, promoted apoptosis in HCT116 and LOVO cells.

Phosphorylation of PI3K activates phosphorylation of AKT and mTOR, which play key roles in regulating cell proliferation, growth, angiogenesis, migration, and nutrient metabolism (Manning and Toker, 2017). Previous studies have shown that numerous natural substances have the ability to inhibit the PI3K/AKT/mTOR signaling pathway in a variety of cancer cells, which is considered to be an key effective strategy for cancer inhibition (Narayanankutty, 2019) and has become a popular target for new cancer drugs (Janku et al., 2018). We detected the phosphorylation level of PI3K/AKT/mTOR pathway members using western blotting and found that ASP inhibited phosphorylation of related proteins in a dose-dependent manner.

Activation of the PI3K/AKT pathway increases the expression of mTOR, which enters the nucleus and initiates the expression of CyclinD1, facilitating the rapid entry of cells into mitosis (Xie et al., 2021). C-Myc stimulates cell cycle progression by regulating many genes related to cell cycle control (Elbadawy et al., 2019; García-Gutiérrez et al., 2019). Claassen et al. (Claassen and Hann, 2000) found that upregulation of p21^CIP1^ correlated with the downregulation of c-Myc levels after transforming growth factor β treatment in HaCaT cells. In the present study, the analysis of the above-mentioned cell cycle proteins revealed that different concentrations of ASP extracts decreased the levels of CyclinD1 and c-Myc proteins and increased the levels of p21 in cells in a concentration-dependent manner. This result indicates that ASP negatively regulates cell cycle proteins in CRC cells.

The role of p53 proteins in apoptosis, senescence, and DNA damage repair is well understood (Lacroix et al., 2020). Bcl-2 family proteins are involved in apoptosis through various mechanisms, and elevated Bax expression is usually associated with apoptosis, while decreased Bcl-2 expression levels indicate a reduced ability to inhibit apoptosis (Ramesh and Medema, 2020), which is closely related to the PI3K pathway (Wang Y. et al., 2020). Cheng et al. (2020) found that naringin effectively promoted Bax expression and inhibited Bcl-2 expression in a concentration-dependent manner. Similarly, our results showed that the pro-apoptotic regulators TP53 and Bax were upregulated and the anti-apoptotic regulator Bcl-2 was downregulated by ASP. The expression of these regulators confirms the pro-apoptotic effect of ASP on CRC. Flow-cytometry apoptosis analysis and cellular TUNEL assays also showed that ASP induced apoptosis in HCT116 and LOVO cells. In addition, it has been suggested that mTOR pathway inhibition might abrogate tumor cell invasion and migration (Duan et al., 2018). The results of our scratch healing and Transwell invasion assays showed that ASP inhibited the migration and invasion of CRC cells.

Based on these results, we found that ASP inhibited cell proliferation, migration and invasion, promoted cell G0/G1 phase block and apoptosis, and inhibited the PI3K/Akt/mTOR pathway to achieve therapeutic effects on colorectal cancer. Although we demonstrated the anticancer effect of ASP and its mechanism, further study is required to determine the mechanism of the antimetastatic effect of ASP on CRC and further *in vivo* experiments are needed to better validate the predicted results of network pharmacology.

## 5 Conclusion

In conclusion, the pharmacological mechanism of ASP’s effect on CRC was explored through a combination of network pharmacological analysis and *in vivo* and *in vitro* experimental validation. We demonstrated that ASP inhibited the proliferation of HCT116 and LOVO cells through PI3K/AKT/mTOR-mediated pathway and induced cell cycle arrest and apoptosis. In addition, ASP reduced the migration and invasion of CRC cells. This study provides a theoretical and experimental basis for the use of traditional Chinese medicines as antitumor agents.

## Data Availability

The original contributions presented in the study are included in the article/Supplementary Material, further inquiries can be directed to the corresponding authors.

## Supplementary Material

Supplementary Figure S1
**(A)** LO2 cells were treated with asparagus water extract (ASP) at the specified concentration for 24 h. **(B)** Cytotoxicity of ASP to LO2 cells in vitro. **P* < 0.05, ***P* < 0.01 vs. control.
