# Supplementary figures and images for: Combining Network Pharmacology and Experimental Validation to Study the Action and Mechanism of Water extract of Asparagus Against Colorectal Cancer
Source: Front Pharmacol. 2022 Jun 14;13:862966. doi: 10.3389/fphar.2022.862966 (PMC9237230; doi:10.3389/fphar.2022.862966)

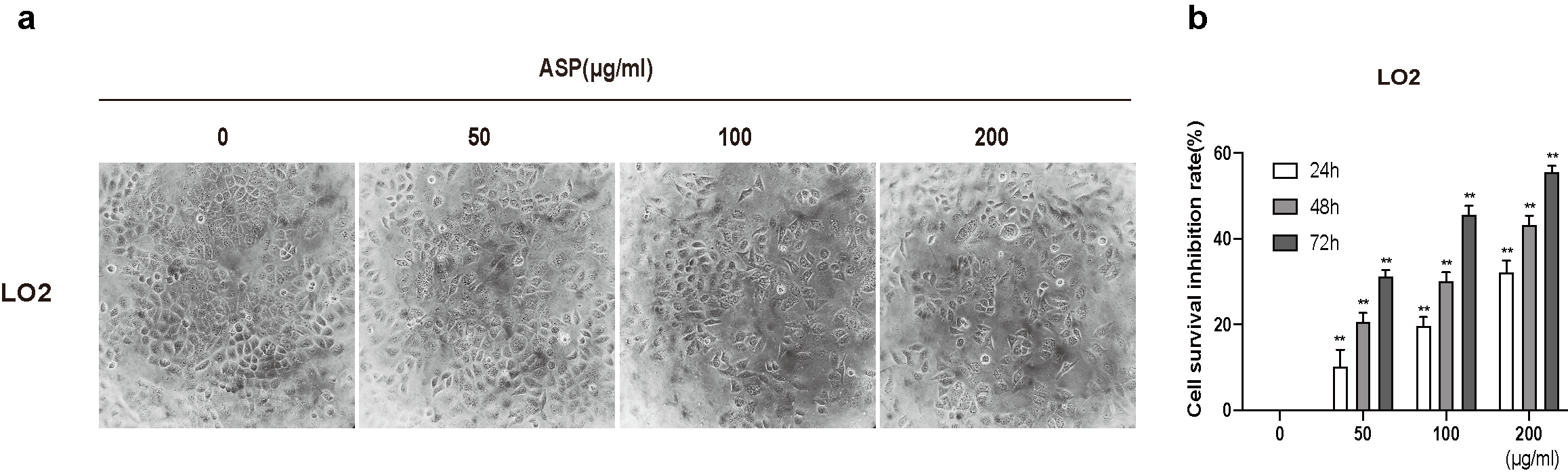

Supplement: Supplementary file 1 [file Image1.tif]
